# Associations of dietary inflammatory index, serum levels of MCP-1 and body composition in Iranian overweight and obese women: a cross-sectional study

**DOI:** 10.1186/s13104-020-05390-x

**Published:** 2020-11-23

**Authors:** Nasim Ghodoosi, Atieh Mirzababaei, Elahe Rashidbeygi, Negin Badrooj, Seyedeh Forough Sajjadi, Leila Setayesh, Mir Saeed Yekaninejad, Seyed Ali Keshavarz, Farideh Shiraseb, Khadijeh Mirzaei

**Affiliations:** 1 Department of Community Nutrition, School of Nutritional Sciences and Dietetics, Tehran University of Medical Sciences (TUMS), P.O. Box: 14155-6117, Tehran, Iran; 2 Department of Epidemiology and Biostatistics, School of Public Health, Tehran University of Medical Sciences (TUMS), Tehran, Iran; 3 Department of Clinical Nutrition, School of Nutritional Sciences and Dietetics, Tehran University of Medical Sciences, Tehran, Iran

**Keywords:** Dietary inflammatory index, Overweight, Obesity, Body composition, Body fat mass, Fat-free mass, MCP-1

## Abstract

**Objective:**

Although, several studies have illustrated that there is a relation between dietary inflammatory index (DII) with obesity-related parameters, and inflammation, their results were controversial. This study aimed to investigate this relationship among Iranian women.

**Results:**

Multivariable linear regression showed that fat mass was 0.14 kg lower in the anti-inflammatory diet group, with respect to the pro-inflammatory group, after adjusting covariates such as age, physical activity, economic and job status (β = − 0.142, 95% CI − 4.44, − 1.71, *P* = 0.03). Fat-free mass (FFM) was 1.5 kg more in the anti-inflammatory diet group, compared to the pro-inflammatory diet group, after adjusting for potentials cofounders (β = 1.50, 95% CI 0, 3.01, *p* = 0.05). Furthermore, after adjusting for potentials cofounders, it was revealed that the subjects with lower DII had lower monocyte chemoattractant protein-1 (MCP-1) levels in serum (β = − 18.81, 95% CI − 35.84, − 1.79, *p* = 0.03). These findings suggest an inverse and significant relationship between DII and FFM and also DII is directly related to Fat mass and the level of MCP-1. This finding can be used for developing interventions that aim to promote healthy eating to prevent inflammation and non-communicable disease development among obese females.

## Introduction

Over the past 3 decades, the prevalence of obesity has nearly doubled worldwide [1]. The mean Body mass index(BMI) for women rose by 0.5 kg/m^2^ per decade from 1980 to 2008 globally [2]. Notably, it has been suggested that more than 21% of women will be obese by 2030 internationally [3]. Obesity results in inflammation cascade [4] and increased the risk of cardiovascular disease (CVD), hypertension (HTN), and stroke [5-8].

Obesity is contributed to low-grade systemic inflammation however, the exact underlying molecular pathway did not understand completely. It has been proposed, however, that this inflammation is created by pro-inflammatory cytokines produced by infiltrated macrophages in adipose tissue [9–11]. The underlying mechanism can be explained by penetration macrophage into adipose tissue, some chemotactic molecules such as monocyte chemoattractant protein-1 (MCP-1), are secreted by white adipose tissue (WAT) [12]. MCP-1 is known as the main ligand of chemokine receptor-2 (CCR2) [13] That plays an important role in obesity-induced inflammatory responses [14]. MCP-1 levels have been related to many diseases, such as CVD, diabetes [15–17]. Recent studies showed that MCP-1 levels can be decreased by an anti-inflammatory diet, such as a Mediterranean diet[18, 19].

Based on the consumption of 45 specific foods and nutrients, dietary inflammatory index (DII) demonstrates the serum levels of six inflammatory biomarkers [Interleukin1beta (IL-1β), Interleukin 4 (IL-4), Interleukin 6 (IL-6), Interleukin 10 (IL-10), Tumor necrosis factor-alpha (TNFα), and C-reactive protein (CRP)] [20]. Moreover, many studies revealed that healthy and unhealthy dietary index such as a vegetarian diet is associated with anthropometric measures or body composition. [21–24]. To our knowledge, a limited number of studies have investigated the relationship between DII, body composition, and serum MCP-1 nevertheless, this association had not been evaluated in Iran. Consequently, the present study decided to assess the relationships between DII with MCP1 and body composition.

## Main text

### Methods

#### Study design and population

A cohort of 280 non-postmenopausal and healthy women aged 18≤ years, who referred to health centers in Tehran, Iran, in 2018 was recruited in this cross-sectional study. Multistage cluster random sampling method was used to select certain regions from among all the regions of the city; finally, 20 clusters were chosen to select the obese and overweight participants (BMI: 25–40 kg/m^2^). The exclusion criteria were as follows: a history of any acute or chronic diseases such as hypertension, CVD, diabetes mellitus, hepatic or renal disease or alcohol consumption, regular usage of medicine other than birth control pills, pregnancy, or lactation. They were excluded if adhered to special dietary patterns or had any significant body weight fluctuations over the past year.

#### Dietary measurements & DII calculation

Dietary assessment was carried out by a validated and reliable 147-item semi-quantitative food frequency question (FFQ) designed according to the Willett study that administered by a trained nutritionist to assess the average daily intake for last year [25]. The FFQ consisted of a list of foods with standard serving sizes. Participants were asked to report their frequency and the amount of each food item consumed during the previous year on a daily (e.g., bread), weekly (e.g., rice, meat), or monthly (e.g., fish) basis. Household measures were used to convert portion sizes of the consumed foods into grams [26], and an estimated average daily intake of food parameters was calculated from the FFQ using NUTRITIONIST IV software (version 7.0; N-Squared Computing, Salem, OR). FFQ-derived dietary data were used to calculate DII scores for all participants. The dietary data were linked to the regionally representative world database that included food consumption from eleven populations around the world and provided a robust estimate of a mean and standard deviation for each parameter [27]. In order to get z-scores, the "standard global mean" was subtracted from the actual dietary intake amount, and this value was divided by the standard deviation. Subsequently, to minimize the effect of ‘right skewing’, these z-scores were converted into a percentile – each percentile score was doubled and then subtracted by 1. The centered percentile score for each food parameter for each individual was then multiplied by the respective food parameter effect score, to obtain a food parameter-specific DII score for an individual [27]. Subsequently, all of the food parameter-specific DII scores were summed together to calculate the overall DII score. Higher DII scores indicated a more pro-inflammatory diet; whereas lower values represented more anti-inflammatory diets [27]. A total of 29 food parameters were available from the FFQ, were used to calculate DII (namely: energy, carbohydrate, protein, total fat, monounsaturated fat, polyunsaturated fat, saturated fat, omega-3, omega-6 fatty acids, cholesterol, fiber, thiamin, riboflavin, niacin, vitamin B6, folic acid, vitamin B12, iron, magnesium, selenium, zinc, β carotene, vitamin A, C, D, E and tea, onion, caffeine).

#### Biochemical assessment

All Biochemical analyses were carried out on venous blood samples that were collected after 12 h fasting, and the serum was centrifuged, liquated, then stored at – 80 °C. Serum MCP-1 levels were measured by the enzyme-linked immunosorbent assay (ELISA) method with an appropriate kit (Zell Bio GmbH, ULM, Germany, assay range: 5 ng/L-1500 ng/L, sensitivity: 2.4 ng/L, inter-assay variability: CV<12%, intra-assay variability: CV<10%).

#### Anthropometric assessment

Bioelectrical impedance analysis (BIA) (InBody 720, Korea) was utilized to calculate body composition measures, including body fat mass and fat-free mass. Anthropometric measures such as body weight, BMI, waist circumferences (WC), and waist-hip ratio (WHR) were measured for all participants. Height was characterized while the subjects were in a standing position without shoes, in contact with the wall with their head, shoulders, heels, and hips, and their height was recorded to the nearest 0.1 cm with Seca 206. All of the measurements were done by expert trained technicians and based on specific guidelines, in order to reduce interpersonal variation.

#### Physical activity assessment

The required information on physical activities in three levels of mild, moderate, and vigorous for research purpose was achieved by using The International Physical Activity Questionnaire (IPAQ) which can be applied by young and middle-aged adults (18–65years). All these sub-components were summed across MET scores and MET-minutes per week (MET-min/wk) were computed and then the total physical activity from all activity categories was reported [28].

### Statistical analyses

DII (dichotomous) was examined across the quantitative characteristics. These were analyzed through an independent sample T-test. Linear regression analyses were conducted to determine the relationship between DII score with fat-free mass and serum MCP-1 levels, adjusted for potential confounding factors. The results are reported as a percentage change (β), with 95% confidence intervals (95% CI). P values of <0.05 were considered to be statistically significant. Statistical analysis was performed using SPSS version 21 (SPSS Inc., Chicago, USA).

### Results

#### Study population characteristics

The DII score in this study ranged from − 4.14 (most anti-inflammatory score) to 3.89 (most pro-inflammatory score). DII was categorized based on the median value of the DII (0.05). A total of 280 females were categorized, based on the DII level, into the anti-inflammatory diet (DII ≤ 0.05) and the pro-inflammatory diet (DII > 0.06) groups. Participant characteristics by DII categories are provided in Table 1. There were no significant differences observed in participant characteristics across the two DII groups. Notably, there were significant differences in fat-free mass (*P* = 0.021) among DII group members and MCP-1 serum levels and physical activity among the DII categories (*P* < 0.0001).

**Table 1:** Participant characteristics and body composition by DII Level

Continuous variables (mean ± SD)	Anti-inflammatory diet	Pro-inflammatory diet	P value
Age (year)	37.60 ± 7.58	35.03 ± 8.48	0.105
Weight (kg)	81.09 ± 12.56	80.61 ± 12.09	0.317
Height (cm)	161.25 ± 6.24	161.33 ± 5.75	0.217
BMI (kg/m^2^)	30.94 ± 3.99	30.52 ± 3.56	0.130
Waist circumference (cm)	99.25 ± 10.24	98.97 ± 9.89	0.391
Body fat mass (kg)	33.98 ± 8.55	34.27 ± 8.89	0.858
Visceral fat area(cm^2^)	162.39 ± 95.08	175.145 ± 10.75	0.307
Fat-free mass (kg)	47.6 ± 20.03	46.5 ± 49.20	0.021
Physical activity (MET h/day)	1162/45 ± 1322/88	773/24 ± 692/04	< 0.0001
MCP-1	30.76 ± 42.01	51.72 ± 67.13	< 0.0001

n = 280

Data are presented as mean ± standard deviation

Nutrients intake adjusted for energy intake before calculating DII

DII values were categorized according to the median (anti-inflammatory diet: DII ≤ 0.05, pro-inflammatory

diet: DII > 0.06)

Independent sample t-test was used for comparison of continuous variables between DII categories

DII food parameters intakes across the anti-inflammatory and pro-inflammatory diet are shown in Table 2. No significant differences were noted regarding energy intakes between DII groups (Table 2). However, among DII groups, some nutrient intakes, such as total fat (*P* = 0.013), saturated fatty acid (SFA), polyunsaturated fatty acid (PUFA), monounsaturated fatty acid (MUFA), omega-6 fatty acids, and Vitamin E (*P* < 0.0001) was higher with those in the pro-inflammatory group, whereas riboflavin (*P* = 0.021), Vitamin C (*P* = 0.031) and Beta-carotene (*P* < 0.0001) were lower intakes mean in the pro-inflammatory diet group compared to the anti-inflammatory diet group (Table 2).

**Table 2:** DII food parameters intake according to DII group

	Anti-inflammatory diet		Pro-inflammatory diet		*P* value
	Mean SD		Mean SD		
Energy intake (kcal)	2588.82	707.15	2658.21	790.42	0.140
Carbohydrate (g)	386.41	39.61	357.23	49.32	0.108
Protein (g)	93.67	14.83	83.12	17.38	0.584
Total fat (g)	87.28	15.56	101.38	22.02	0.015
MUFA (g)	29.01	6.52	33.55	10.42	< 0.0001
PUFA (g)	18.85	5.70	21.23	9.40	< 0.0001
SFA (g)	25.98	6.05	30.13	8.55	< 0.0001
Cholesterol (mg)	256.77	79.80	248.50	94.54	0.113
Fiber (g)	50.09	12.70	40.34	14.21	0.439
Thiamine (mg)	2.13	0.31	2.01	0.39	0.156
Riboflavin (mg)	2.31	0.43	2.06	0.64	0.023
Niacin (mg)	26.49	4.88	23.92	6.85	0.165
Vitamin B6 (mg)	2.38	0.37	1.92	0.38	0.354
Folic acid (µg)	648.31	101.31	562.32	92.69	0.222
Vitamin B12 (µg)	4.88	1.88	4.55	2.33	0.402
Vitamin A (RE)	931.58	369.19	616.03	287.89	0.044
Vitamin C (mg)	257.56	98.48	172.09	106.78	0.024
Vitamin E (mg)	16.17	5.92	18.52	10.85	< 0.0001
Vitamin D (µg)	2.84	1.45	2.45	1.63	0.943
Iron (mg)	19.79	2.44	17.43	2.83	0.431
Beta-carotene (µg)	6910.62	3652.64	3581.06	1912.90	< 0.0001
Selenium (µg)	123.90	28.14	115.70	30.25	0.542
Zinc (mg)	13.71	1.97	12.03	2.26	0.214
Magnesium (mg)	510.30	68.62	404.72	66.85	0.811
Caffeine (g)	205.50	189.42	167.97	96.51	0.058
Omega 3 (g)	1.39	0.56	1.35	0.65	0.513
Omega 6 (g)	15.98	5.46	18.67	9.24	< 0.0001
Onion(g)	33.82	23.44	30.97	21.04	0.498
Tea(g)	963.42	946.41	795.08	489.88	0.082

n = 280

Data are presented as mean ± standard deviation

Comparisons of nutrient intake across the groups of the DII were analyzed using independent sample t-test

DII values were categorized according to the median (anti-inflammatory diet: DII ≤ 0.05, pro-inflammatory

diet: DII > 0.06)

Nutrients intake adjusted for energy intake

Results obtained from modeling DII as a dichotomous variable in relation to the fat-free mass showed a negative association and positive association between DII adherence and fat-free mass and fat mass respectively, after adjustment for age, physical activity, economic and job status (*P* = 0.05, *P* = 0.03) (Table 3). As shown in Table 3, the fat-free mass was 1.5 kg more in the anti-inflammatory diet group, compared to the pro-inflammatory diet group, with adjusted confounders (β = 1.50, 95% CI 0, 3.01, *P* = 0.05), fat mass was 0.14 kg lower in the anti-inflammatory diet group, with respect to the pro-inflammatory group, with adjusted covariates (β = − 0.142, 95% CI − 4.44, − 1.71, *P* = 0.03). Linear regression tests demonstrated a positive relationship between DII and serum MCP-1 level after adjusting for confounders (*P* = 0.03). MCP-1 serum levels was 18.81 lower in the anti-inflammatory diet group, with respect to the pro-inflammatory group, with adjusted covariates (β = − 18.81, 95% CI − 35.84, − 1.79, *P* = 0.03) (Table 3).

**Table 3:** The Association between DII, Fat-Free Mass, Fat Mass, and MCP-1

Variables		Β	95% (CI)	*P* value
Fat free mass				
Anti-inflammatory diet		1.50	(0.01,3.01)	0.050*
Pro-inflammatory diet		Ref.	Ref.	
Fat mass				
Anti-inflammatory diet		− 0.142	(− 4.44,− 0.171)	0.034*
Pro-inflammatory diet		Ref.	Ref.	
MCP-1				
Anti-inflammatory diet		− 18.81	(− 35.84,− 1.79)	0.030**
Pro-inflammatory diet		Ref.	Ref.	

n = 280

DII values were categorized according to the median

(Anti-inflammatory diet: DII ≤ 0.05, Pro-inflammatory diet: DII > 0.06)

*P* value*: adjusted for age, physical activity, economic and job status

*P *value **: adjusted for age, weight, smoking, physical activity, economic and job status

## Discussion

The current study set out to examine the associations between dietary inflammatory potential, body composition, and inflammation in Iranian obese women. Previous studies have shown that different dietary components have varying effects on body composition, and inflammation. In this cross-sectional study, it was found that subjects with a higher DII had lower fat-free mass and higher fat mass than others, independent of potential confounders. Furthermore, independent of confounders, the DII, and MCP-1 are related.

A dissident study in Spain demonstrated that a higher DII score was inversely associated with obesity-related parameters such as FFM and weight [29]. The previous papers have shown DII was related to higher average BMI, WC, and WHR [23, 30, 31]. The associations between fat mass, BMI, and waist circumference with inflammatory molecules such as IL-1, IL-6 have been suggested [32, 33]. In this context, increased circulating cytokine levels, have been described in relation to a decrease in FFM [34]. A possible mechanism was theorized that a diet with an inflammation-induced potential is correlated with higher inflammatory response, and may result in lower fat-free mass. Furthermore, individuals with higher adherence to DII had a lower intake of some nutrients like vitamins C and E, magnesium, potassium, and a range of carotenoids such as Beta carotene that the amount of intake of these nutrients was positively associated with muscle mass in women. Moreover, based on previous evidence, higher adherence to the healthy dietary pattern as the Mediterranean Diet, Healthy Diet Indicator, Diet Quality Index, Alternate Healthy Eating Index, and DASH-style was significantly associated with measurements of muscle mass. Consumption of a variety of plant-based nutrients and overall higher quality in some dietary patterns resulted in the conservation of muscle mass [35]. M. Ruiz-Canela et al. shows participants with higher pro-inflammatory diet also had higher BMI and WHR [36]. Some potential mechanisms are hypothesized, such as activation of pathogen-associated molecular patterns, such as nod and toll-like receptors (TLRs), that induce the activation of inflammatory markers particularly in adipose tissues [37]. Also, intestinal microbiota may be affected by high-fat or low-fiber dietary patterns, which also appear to be associated with low-grade inflammation and obesity [38–40]. Evidence suggests that weight gain can be predicted by the proteins in plasma that are sensitive to inflammation [41]. Serine phosphorylation is another conceivable mechanism that assumes a function in inflammation. Inflammation causes phosphorylation of both insulin receptor and its proteins substrate, including the enzyme phosphatidylinositol 3-OH kinase (PI3 K) bind together, then the inflammatory pathway, mainly I kappa B kinase (IKKb) and Nuclear factor-kappa B (NF-kB) pathway will be activated, finally inhibits insulin-mediated PI3K signaling [42]. IKKb/NF-kB activation also blocked cytokine signaling-3, a protein that blocks cell signals from insulin [43, 44]. Ultimately, these processes trigger suppression of appetite [45].

This study was indicated that participants with higher DII had a greater level of MCP-1. In a study conducted in Belgium was seen that DII and inflammatory markers IL-6 have been associated. Although there were no significant associations between CRP and fibrinogen [46]. Moreover, Shivappa et al. showed that pro-inflammatory diets were related to increased levels of various inflammatory markers such as TNF-α, IL-1, 2, IFN-γ, and vascular cell adhesion molecules [47]. The research was conducted in Spain demonstrated that an anti-inflammatory diet, such as a Mediterranean diet, was associated with a significant reduction of IL-6, IL-8, and MCP-1 [48]. Therefore, one possible mechanism seems to suggest that anti-inflammatory diets can modify inflammatory responses, is due to their antioxidants, fiber content, and other anti-inflammatory substances [19, 48].

To the researchers’ knowledge, this is the first study conducted which examines the association between dietary inflammatory potential, body composition, and MCP-1 levels.

## Conclusion

The present results provide evidence supporting the proposition that a higher DII score (pro-inflammatory diet) is directly associated with lower fat-free mass, higher fat mass, and higher levels of inflammatory markers like MCP-1. These results suggest the importance of promoting dietary patterns with low inflammatory potential to reduce low-grade inflammation and improve obesity- parameters among the overweight and obese population.

## Limitations

This study has several limitations. First, include the reliability and validity of the estimation of average food intakes, which were based on the relatively limited number of food items (147 items, which is moderately long for an FFQ). Second, the DII was calculated using the data on just 29 food parameters derived from the FFQ. Third, causality cannot be inferred, because of the cross-sectional design of the study. Fourth, it is not possible to generalize about dietary patterns throughout the country, because dietary intakes and other lifestyle measurements in Tehran are somewhat different from those in other parts of the country. Moreover, these dietary patterns were confined to women.

## Data Availability

Participants of this study did not agree for their data to be shared publicly, so supporting data is not available.

## Abbreviations

AHEI: Alternate Healthy Eating Index
BMI: Body mass index
CVD: Cardiovascular disease
CCR2: Chemokine receptor-2
CRP: C-reactive protein
DII: Dietary inflammatory index
DQI: Diet Quality Index
ELISA: Enzyme-linked immunosorbent assay
FFQ: Food frequency questionnaire
FM: Fat mass
FFM: Fat-free mass
HTN: Hypertension
Hs-CRP: High-sensitivity C-reactive protein
HDI: Healthy Diet Indicator
IPAQ: International Physical Activity Questionnaires
IFN-γ: Interferon-gamma
IL-2: Interleukin-2
IL-4: Interleukin-4
IL-6: Interleukin-6
IL-8: Interleukin-8
IL-10: Interleukin-10
IL-1β: Interleukin-1β
IKKb: I kappa B kinase
MCP-1: Monocyte Chemoattractant Protein-1
MUFA: Monounsaturated fatty acids
METs: Metabolic Equivalent Tasks
NF-kB: Nuclear factor kappa B
PUFA: Polyunsaturated fatty acids
PI3 K: Phosphatidylinositol 3-OH kinase
SFA: Saturated fatty acids
TNFα: Tumor necrosis factorα
TC: Total cholesterol
TG: Triglycerides
WHO: World Health Organization
WHR: Waist to hip ratio
WAT: White adipose tissue
